# Polyvinyl Alcohol Hydrogel Hemiarthroplasty of First Metatarsophalangeal Joint Hallux Rigidus

**DOI:** 10.7759/cureus.58583

**Published:** 2024-04-19

**Authors:** Albert T Anastasio, Aman Chopra, Naji M Madi, Troy Q Tabarestani, Amanda N Fletcher, Selene G Parekh

**Affiliations:** 1 Department of Orthopedic Surgery, Duke University Medical Center, Durham, USA; 2 Department of Orthopedic Surgery, Georgetown University School of Medicine, Washington, DC, USA; 3 Department of Neurosurgery, Duke University School of Medicine, Durham, USA; 4 Department of Orthopaedic Surgery, Rothman Orthopaedic Institute, Philadephia, USA

**Keywords:** metatarsophalangeal joint, hydrogel implants, polyvinyl alcohol, hemiarthroplasty, hallux rigidus

## Abstract

Background

Hallux rigidus (HR) is the most common arthritic condition of the foot. Although first metatarsophalangeal joint (MTPJ) arthrodesis has been the historical gold-standard treatment, polyvinyl alcohol (PVA) hydrogel implants have gained popularity as a joint-sparing technique. However, recent studies have shown variable failure rates of PVA hydrogel implants. The purpose of this study was to report the five-year experience with PVA hydrogel implants performed by a single surgeon.

Methodology

Health records were queried from August 2016 to 2021 for patients who underwent primary PVA hydrogel implant hemiarthroplasty for symptomatic late-stage HR. Patient demographics and postoperative outcomes variables were evaluated. Kaplan-Meier analysis was used to evaluate implant survival. A total of 146 PVA hydrogel implant procedures were performed with a minimum six-month follow-up.

Results

The majority of patients were female (n = 103, 70.5%), with a mean age of 58.1 (±10.1) years, body mass index of 27.3 (±5.2) kg/m^2^, and American Society of Anesthesiologists score <3 (n = 131, 89.7%). The majority had stage II or III disease (n = 115, 78.8%). Patients experienced significant improvement in visual analog scale score (p < 0.0001) and hallux dorsiflexion (p = 0.0005). There were 22 (15.1%) complications, including implant subsidence (n = 15, 10.3%), deep infection (n = 6, 4.1%), and hypertrophic ossification (n = 1, 0.7%). Revision surgeries were required in 12.3% (n = 18) of patients at an average of 9.4 (±9.2) months postoperatively. This included nine (6.2%) revision PVA hydrogel implant procedures and nine (6.2%) first MTJP arthrodesis. The one- and two-year survival rates for MTPJ arthrodesis (n = 9) were 95.9% and 86.3%, respectively.

Conclusions

In the largest single-surgeon series reported, first MTPJ hemiarthroplasty with a PVA hydrogel implant significantly improved pain and hallux dorsiflexion at an average of 14.5 months postoperatively. There was a high two-year survivorship of 86.3% until failure which required first MTPJ arthrodesis. Future studies should be performed to refine the indications for PVA hydrogel implants and identify risk factors.

## Introduction

Hallux rigidus (HR), or severe first metatarsophalangeal joint (MTPJ) arthritis, is a condition characterized by pain and reduced range of motion to the first metatarsal phalangeal joint [1]. It is currently recognized as the most common arthritic condition affecting the foot with a prevalence of up to 45% in people aged over 75 years [2]. Treatment of this condition includes nonoperative and operative techniques, with nonoperative measures primarily aimed at shoe wear and lifestyle modification [3]. Given the rising demand for operative treatment of HR in an aging population, a variety of surgical techniques have been developed for the treatment of severe MTPJ arthritis [4-10]. These treatment modalities can be largely subdivided into joint-sparing and joint-sacrificing techniques.

Regarding joint-sacrificing techniques, metatarsal phalangeal joint arthrodesis is the gold-standard treatment for late-stage HR, with excellent improvement in patient-reported outcomes and pain scores [11]. However, arthrodesis compromises the normal biomechanical function of the foot and can be a displeasing option for some patients. Thus, recent emphasis on joint-sparing procedures has led to the development of a variety of techniques beyond dorsal cheilectomy. These techniques include various forms of interpositional arthroplasty [12-14]. Survivorship rates of these implants, however, are variable [15]. Revision to metatarsophalangeal arthrodesis after an attempt at interpositional arthroplasty is fraught with complications, demonstrating higher rates of nonunion and the need for revision compared to primary arthrodesis [15].

Given the variable clinical outcomes and survivorship rates of existing forms of interpositional arthroplasty, there remains room for improvement in current joint-sparing techniques for HR. A Synthetic Cartilage Implant (SCI) was created to mimic the biomechanical properties of normal human cartilage with the goal of more closely replicating native foot mechanics than previous interpositional products [16]. SCI measures 8 mm, 10 mm, or 12 mm in diameter × 10 mm in depth and is manufactured from polyvinyl alcohol (PVA) [17]. To date, there is little literature analyzing the outcomes and survival rates of SCI. An existing randomized control trial, the Cartiva Motion Study, is an industry-funded analysis that demonstrated improvements in patient-reported functionality and pain reduction at the two and five-year time points [18]. Regardless, additional, nonindustry-funded studies are indicated.

The purpose of this study, therefore, is to report complication rates, survival rates, and improvements in pain scores and the first MTPJ range of motion after SCI for HR. Our study represents the largest single-surgeon study published to date.

This article was previously presented as a meeting abstract at the 2022 American Orthopaedic Foot and Ankle Society Annual Meeting on September 14, 2022.

## Materials and methods

Patient data

After obtaining institutional review board approval (ID: Pro00108507), electronic medical records were retrospectively queried for patients treated with SCI resurfacing (Cartiva Synthetic Cartilage Implant; Wright Medical Group, Memphis, TN, USA) for symptomatic late-stage HR between August 2016 and August 2021. All patients included in the study were over 18 years of age, had a minimum follow-up duration of six months, and were treated by a single foot and ankle fellowship-trained orthopedic surgeon at an academic institution. Exclusion criteria included concomitant hallux valgus with an intermetatarsal angle greater than 13 degrees, patients aged less than 18 years, and patients receiving isolated dorsal cheilectomy.

Patient data was retrospectively collected, including demographics, body mass index (BMI), medical comorbidities, American Society for Anesthesiologists (ASA) physical status, previous surgical history, and concomitantly performed procedures. Using preoperative clinical and radiographic findings, the severity of the first MTPJ HR was classified using the Coughlin and Shurnas Clinical Radiographic Scale [19]. Revision surgery, implant survivorship, and postoperative complications, such as heterotopic ossification, deep infection, and implant subsidence, were recorded. Preoperative and most recent postoperative MTPJ dorsiflexion, plantarflexion, and visual analog scale (VAS) pain scores were obtained by the senior surgeon. Patient-reported outcome scores were unavailable at the time of data collection given heterogeneous collection.

Surgical technique

**Figure 1:**
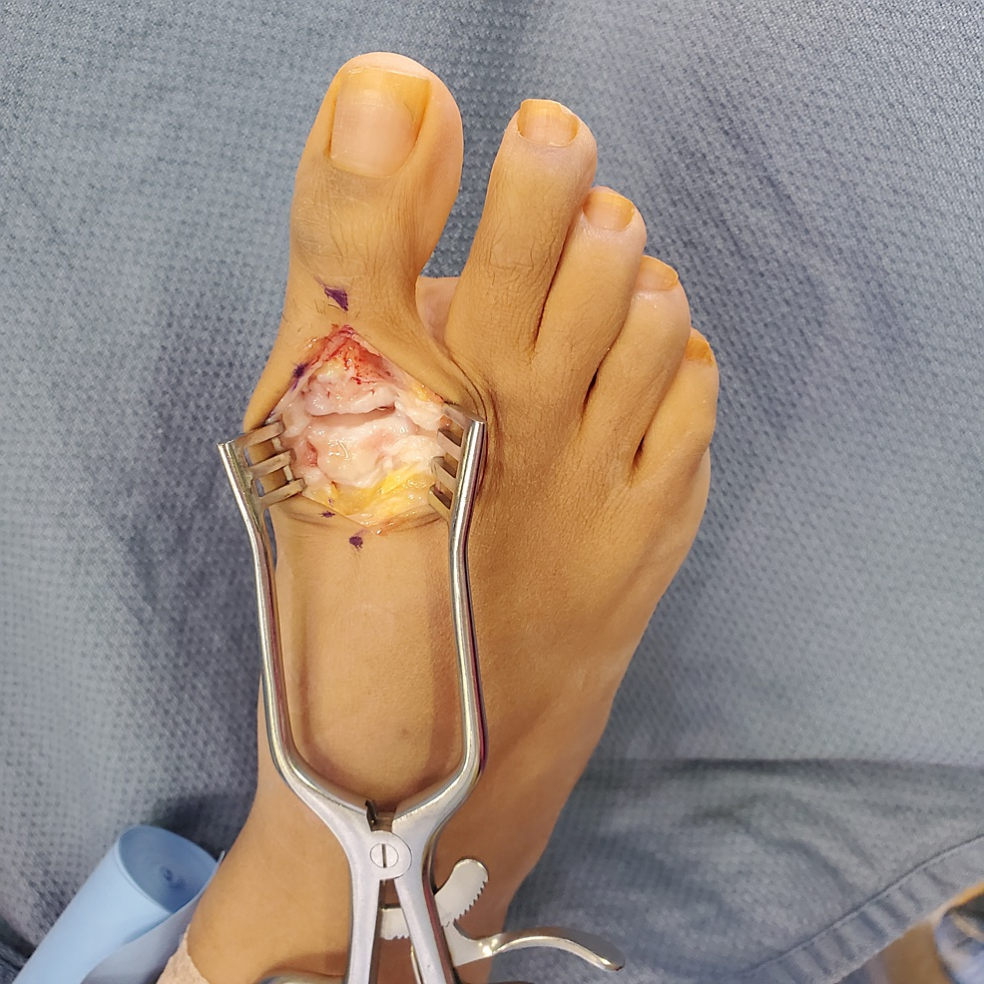
The extensor hallucis longus tendon is retracted using a Weitlaner retractor and a longitudinal capsulotomy is performed to expose the joint.

A longitudinal 5 cm dorsal incision was made over the first MTPJ. The extensor hallucis longus tendon was exposed and retracted laterally and the extensor hallucis brevis was released from its most distal insertions on the proximal phalanx. The joint capsule was opened longitudinally with consideration for surrounding neurovascular structures (Figure 1).

**Figure 2:**
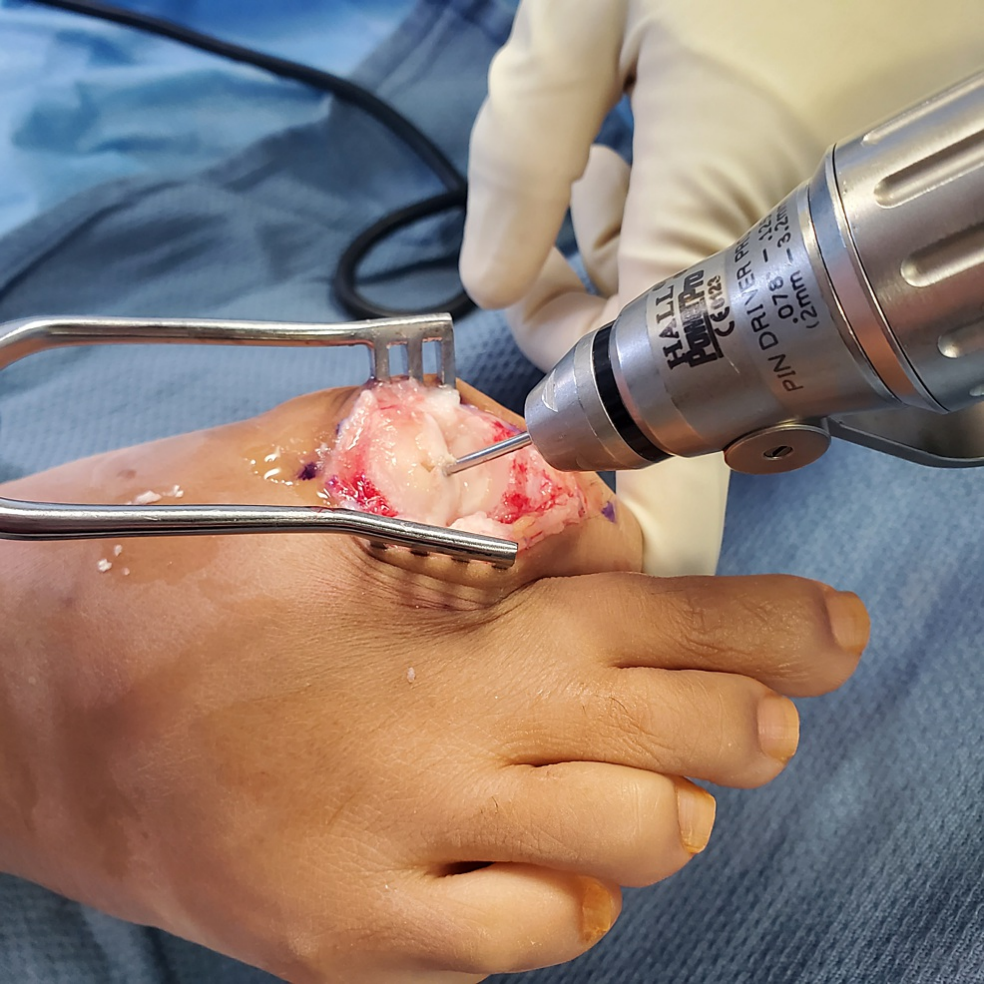
A 2 mm Kirschner wire is inserted centrally in the metatarsal head.

The dorsal third of the collateral ligaments was then released on the metatarsal side and the first MTPJ was maximally plantarflexed to expose the metatarsal head. A saw or small rongeur can be utilized to carefully remove surrounding osteophytes from the metatarsal head. An SCI sizer was then used to determine the appropriate implant diameter (8, 10, and 12 mm sizes are available). The appropriate diameter allows for the largest implant which ensures a remaining 2 mm healthy rim of stable bone or cartilage. A 2.0 mm K-wire was centrally inserted into the metatarsal head with consideration for a minimum of 2 mm cortical rim required to support the implant (Figure 2).

**Figure 3:**
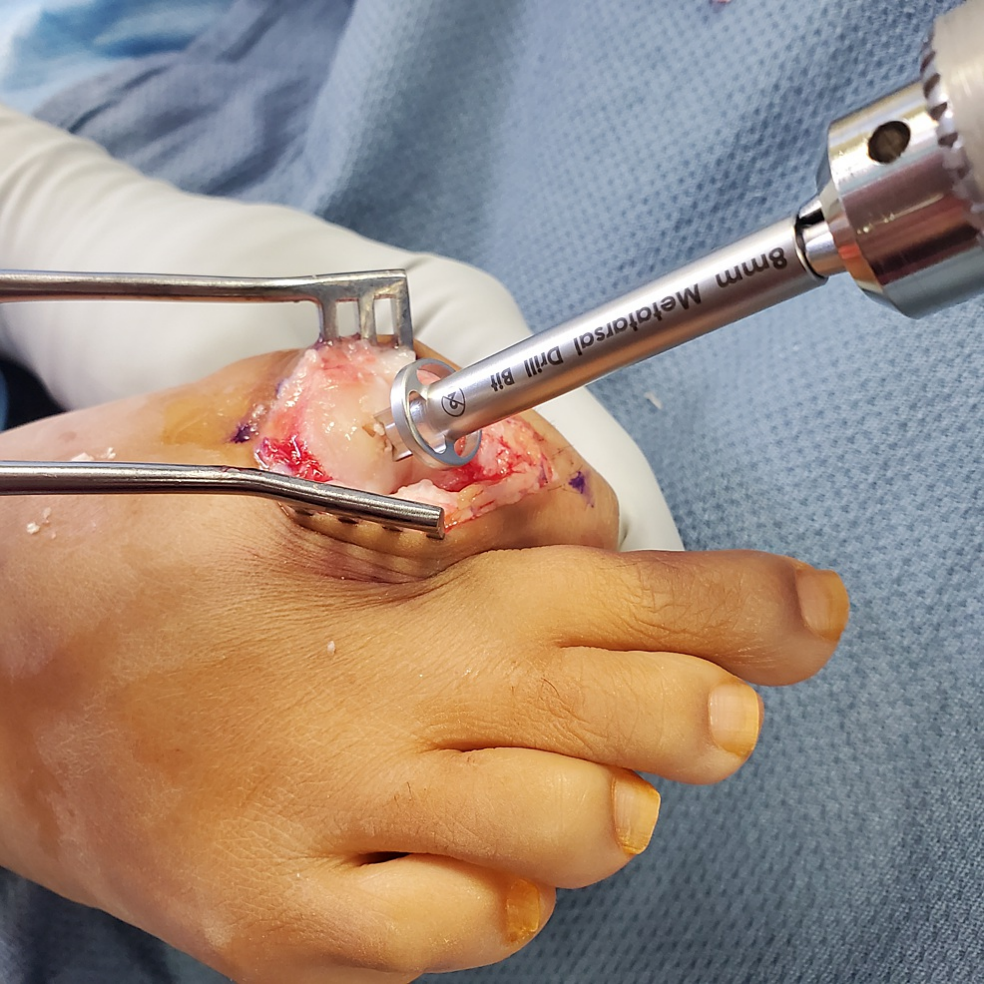
Reaming is performed over the Kirschner wire.

The reamer was placed over the K-wire and reaming was performed (Figure 3).

**Figure 4:**
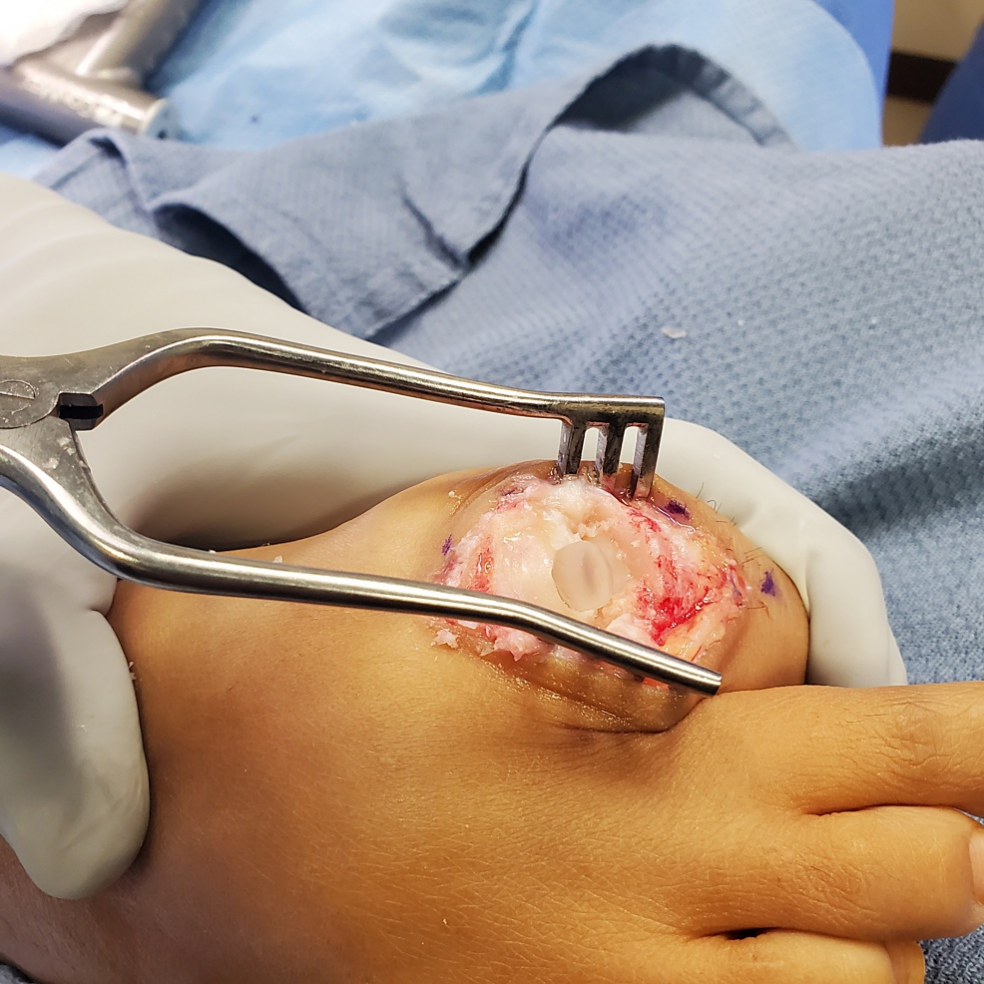
Sagittal view of the inserted Synthetic Cartilage Implant. Note that the implant is appropriately positioned to recreate the joint space but not overly proud to introduce an undue increase in joint contact pressure.

The remaining debris was irrigated out and any loose cartilage around the reamed hole was resected. The SCI was inserted into the loading device and then subsequently pushed into the metatarsal head cavity. The implant position was visually confirmed to be protruding 2 to 3 mm above the metatarsal head (Figure 4).

**Figure 5:**
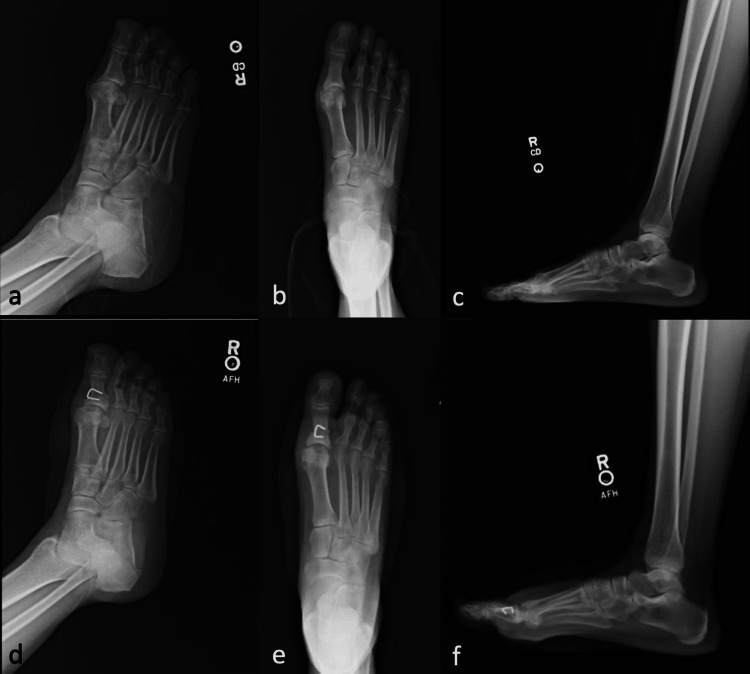
Preoperative oblique (A), anteroposterior (B), and lateral (C) weight-bearing radiographic views of the right foot with hallux rigidus compared with postoperative oblique (D), anteroposterior (E), and lateral (F) weight-bearing views of the right foot following Synthetic Cartilage Implant placement and Akin osteotomy.

After final fluoroscopic images were obtained, the MTPJ capsule and skin were closed in a layered fashion with vicryl suture for the deep layers and silk suture for the skin [20]. Postoperative oblique, anteroposterior, lateral, and weight-bearing films were obtained to confirm the proper placement of hardware (Figure 5).

Statistical analysis

Descriptive and univariate statistics were performed using SPSS Statistics®, version 25.0 (IBM Corp., Armonk, NY, USA) and Microsoft Excel®, version 16.52 (Microsoft Corp., Redmond, WA, USA). Preoperative and postoperative measurements were compared using paired t-tests. A Kaplan-Meier analysis was used to evaluate the one-year and two-year implant survival rates. The Kaplan-Meier survivorship analysis was performed with the revision to arthrodesis as the endpoint for failure. Statistical significance was established at p-values <0.05.

## Results

Over the five-year study period, a total of 196 SCI resurfacing procedures were performed, of which 146 procedures had a minimum six-month follow-up and were included in the final analysis. The mean follow-up duration for the cohort was 14.5 ± 11.9 (range = 6-55) months. The majority of patients were female (n = 103, 70.5%), with a mean age of 58.1 ± 10.1 years and a mean BMI of 27.3 ± 5.2 kg/m^2^. The majority of the cohort’s ASA scores were less than 3 (n = 131, 89.7%). The majority of the patients included in the study had arthritis classified as HR stage 2 (n = 53, 36.3%) and stage 3 (n = 62, 42.5%). The most common procedure performed alongside the SCI resurfacing surgery was an Akin osteotomy (n = 121, 82.9%) of the proximal first phalanx, followed by Weil osteotomies (n = 10, 6.8%) of the lesser toes (Table 1).

**Table 1:** Preoperative demographic data. Values in N (%) or mean (standard deviation; range).

Variables measured	Values
Feet	146
Sex, female	103 (70.5)
Age, years	58.1 (±10.1)
Operative side, right	93 (63.7)
Follow-up, months	14.5 (±11.9)
Body mass index, kg/m^2^	27.3 (±5.2)
American Society of Anesthesiologists (ASA) score < 3	131 (89.7)
Hallux rigidus stage 1	30 (20.5)
Hallux rigidus stage 2	53 (36.3)
Hallux rigidus stage 3	62 (42.5)
Hallux rigidus stage 4	1 (0.7)
Concomitant surgeries: Akin osteotomy	121 (82.9)
Concomitant surgeries: Weil osteotomy lesser toes	10 (6.8)
Concomitant surgeries: Hammer toe correction lesser toes	5 (3.4)
Concomitant surgeries: Removal of hardware	6 (4.1)

The decision to proceed with Akin osteotomy occurs after completion of the SCI procedure. If a patient remains with hallux valgus interphalangeus after the procedure, an Akin osteotomy is performed and fixated with a nitinol staple. The high incidence of Akin osteotomy in this cohort indicates high rates of hallux valgus interphalangeus in our patient cohort receiving the SCI.

In total, 22 (15.1%) patients presented with postoperative complications, which included implant subsidence (n = 15, 10.3%), deep infection (n = 6, 4.1%), and hypertrophic ossification (n = 1, 0.7%). Overall, 18 (12.3%) patients ultimately underwent a revision SCI resurfacing procedure at an average of 9.4 ± 9.2 months (range = 0-32) following the index procedure. Specifically, nine (6.2%) procedures were revised to first MTPJ arthrodesis and nine (6.2%) procedures involved an SCI replacement, of which six (4.1%) procedures included irrigation and debridement. The one- and two-year survival rates for any revision surgery (n = 18) were 89.1% and 80.5%, respectively. The one- and two-year survival rates for MTPJ arthrodesis (n = 9) were 95.9% and 86.3%, respectively (Table 2). Our indications for revision conversion to first MTPJ arthrodesis were symptomatic subsidence of implant and pain refractory to conservative measures. In all cases of refractory pain requiring revision, a degree of subsidence, subluxation, or implant migration was observed.

**Table 2:** Complication, revision, and survival rates. Values in N (%) or mean (standard deviation; range). PVA = polyvinyl alcohol; MTPJ = metatarsophalangeal joint; I&D = irrigation and debridement

Variables measured	Values
Complications: Implant subsidence	15 (10.3)
Complications: Hypertrophic ossification	1 (0.7)
Complications: Deep infection	6 (4.1)
Complications: Total	22 (15.1)
Revision surgery: Revision to PVA hydrogel implant	9 (6.2)
Revision surgery: Isolated revision	3 (2.1)
Revision surgery: I&D with revision	6 (4.1)
Revision surgery: First MTJP arthrodesis	9 (6.2)
Time to first revision surgery	9.4 (±9.2, range 0–32)
Two-year survival: Any revision surgery (n = 18)	80.5%
Two-year survival: First MTJP arthrodesis	86.3%

Patients experienced significant improvement between their preoperative and postoperative pain VAS scores (5.7 ± 1.9 vs. 2.5 ± 2.3, p < 0.0001) as well as first MTPJ dorsiflexion (37.6 ± 29.5 vs. 48.2 ± 26.8, p = 0.0005). No statistically significant improvement was observed for first MTPJ plantarflexion (Table 3).

**Table 3:** Pain and functional improvement metrics. Values in N (%) or mean (standard deviation; range). *: Difference between preoperative and postoperative values.

Variables measured	Preoperative	Postoperative	P-value*
Visual analog scale score	5.7 (±1.9)	2.5 (±2.3)	<0.0001
Hallux dorsiflexion (degrees)	37.6 (±29.5)	48.2 (± 26.8)	0.0005
Hallux plantarflexion (degrees)	12.6 (±11.0)	14.6 (±12.3)	0.2073

## Discussion

HR or first MTPJ arthritis is a significant cause of morbidity and loss of function in the aging population [1]. Given the prevalence of this condition, a vast array of both nonoperative and operative treatment modalities have been developed. Increasing emphasis on joint-sparing techniques has led to the development of a variety of interpositional arthroplasty implants over recent years. The SCI was developed in an attempt to improve upon existing first MTPJ arthroplasty options by replicating normal human cartilage through the use of PVA materials [16]. Our study aimed to address the paucity of literature evaluating outcomes and early survivorship rates of the SCI by reporting the largest single-surgeon cohort published to date.

Our study expands on previous literature demonstrating excellent results of the SCI with statistically significant improvements in VAS pain scores and hallux dorsiflexion. Hallux plantarflexion did not show statistically significant change from preoperative to postoperative values. We report 2-year survivorship rates of 80.5% to any revision surgery and 86.3% to the need for first MTPJ arthrodesis. We demonstrate low complication rates, with implant subsidence as the most commonly occurring complication, with 15 cases in a cohort of 146 SCI for HR.

All results are in congruence with those reported in the Cartiva Motion Study, the originator, industry-funded, randomized controlled trial evaluating the SCI. Across 12 centers in Canada and the United Kingdom, 152 SCI cases were included in the study and were compared against 51 first MTPJ arthrodesis cases as the gold-standard control. VAS pain scores significantly decreased in both groups at the one-year and two-year time points. The authors reported an improvement of 6.2 degrees of MTPJ dorsiflexion, which lags behind our reported dorsiflexion improvement of 10.6. Our senior surgeon attributes special attention to dorsal osteophyte removal and capsular release to the above-average improvement in hallux dorsiflexion demonstrated in our series. Regarding the improvement in dorsiflexion, we also counsel patients preoperatively that while range of motion may improve given resection of arthritic cartilage, joint stiffness often persists. This is because extensive cheilectomy is precluded with the use of the SCI given the need for an adequate rim of bone stock around the implant. As a final point of concordance with the Cartiva Motion Study, patients randomized to the SCI experienced a rate of subsequent secondary surgery of 11.2%, which closely matches our revision surgery rate of 12.3%.

The study by Brandao et al. further corroborates our results in a sample of 55 patients with a mean follow-up of 21 months, the largest single-surgeon sample published before the current study. The study showed improvement in functionality during activities of daily living, as well as improvement in all three Manchester-Oxford Foot Questionnaire domain scores. They reported very low revision rates of only one SCI-to-SCI procedure and one revision for arthrodesis. However, 15 patients did require manipulation under anesthesia and steroid and local anesthetic injection at 12 weeks postoperatively due to stiffness. None of these patients required repeat manipulation or injection. The authors also reported a higher rate of dissatisfaction with the procedure in the female cohort. They attributed this poor satisfaction rate to higher rates of psoriatic arthritis and concomitant sesamoidal arthritis of the female population in the sample. Additionally, they concluded that the higher prevalence of osteoporosis in postmenopausal women may impede the integrity of the press-fit technique of implant introduction into the metatarsal head [20].

In contradiction with our positive outcomes, Cassinelli et al. reported neutral to poor results of the SCI in a single-surgeon sampling of 64 cases of HR [21]. They reported rates of 11% of patients being “unsatisfied” with the procedure and 27% being “very unsatisfied.” There was a relatively high reoperation rate of 20% in this series, and 30% of the patients underwent postoperative magnetic resonance imaging given significant pain. Despite these somewhat negative results, only a slightly higher percentage of patients (8%) required conversion to arthrodesis compared to our cohort (6.2%).

Utilizing the Manufacturer and User Facility Device Experience (MAUDE) database of the Food and Drug Administration, in 2020, Metikala et al. published a descriptive report of the 49 events related to complications of the SCI that had been listed in the database from July 2016 to October 2019 [22]. They reported implant subsidence as the most commonly reported complication with 16 events. Other reported events included fragmentation, infection, bone erosion, foreign body reaction, and unspecified events [1,3,4,9,16]. This analysis disclosed certain device-related malfunctions that have previously been underreported in the literature. However, as event reporting to the MAUDE database is voluntary, this sampling may constitute a poor representation of the actual incidence and subtype of SCI-related complications.

Despite the paucity of studies reporting on the use of SCI for HR, a recent systematic review evaluated the use of SCI for HR [23]. The review concluded that a moderate recommendation can be given for the use of a PVA implant such as SCI for HR based on short-term outcomes. The review provided a limited recommendation for the use of an SCI implant based on midterm outcomes. It did not comment on long-term outcomes, given the lack of available data. Of note, seven studies were included in the systematic review, six of which had been derived from the randomized control trial, the Cartiva Motion Study. The lack of breadth of the studies included in this systematic review may substantially limit the conclusions that can be drawn therein.

To date, there have been no high-quality, randomized controlled trials comparing SCI to other existing interpositional arthroplasty implants such as the Toe HemiCAP® system (Arthrosurface Inc., Franklin, MA, USA) or the ToeFit-Plus® implant (Smith and Nephew, London, UK). However, a randomized controlled trial published in 2016 compared the Toe HemiCAP® to the ToeFit-Plus® showing excellent results for both implants, with statistically significant improvement in range of motion in both groups [24]. Additionally, the study reported improvement in American Orthopaedic Foot and Ankle Society scores as well as VAS pain scores in both cohorts. It did not find a statistically significant difference in any of the outcome metrics between the two implants. It concluded that both implants are effective joint-sparing treatment modalities for HR. Future research should aim to compare SCI directly to other existing interpositional arthroplasty products to determine the optimal choice for patients desiring operative treatment for HR with a joint-sparing procedure.

As a final comment on the indications for SCI in foot surgery, the SCI implant has also been utilized for the treatment of conditions affecting the second metatarsal head, such as Freiberg’s disease and osteochondral defects. Brandao et al. presented a single-surgeon sample comparing six patients receiving SCI and seven patients receiving a primary osteotomy for the pathology of the second metatarsal head at a mean follow-up of 19 months and 27 months, respectively [25]. While the study did report improvements in pain and walking domains after SCI, it reported an extremely high revision rate of four out of six SCI cases. The SCI functioned especially poorly as a treatment for Freiberg’s disease: three of the four revision cases were performed initially for the treatment of this condition. The study concluded that the SCI compares poorly to osteotomy procedures for the treatment of metatarsal head conditions, especially for cases of avascular necrosis of the metatarsal head.

As an important limitation of the present study, our average follow-up was 14.5 months. Thus, while this is the largest single-surgeon sample to date, our outcomes constitute early- to mid-term outcomes. Failure rates such as implant subsidence may be noted at higher rates with longer-term follow-up. We hope to build upon these results by presenting favorable mid- to late-term results, and future research should continue to examine outcomes after SCI, given the substantial heterogeneity in the available data. Additionally, the study methodology of a case series precludes the comparison of SCI with another treatment option. Another limitation was the inclusion of only patients operated on by a single surgeon, which may limit the generalizability of findings. However, in collecting single-surgeon experiences, we provide data for a meta-analysis, which may ultimately provide the answer to controversial treatment options within foot and ankle surgery.

## Conclusions

In the largest single-surgeon series to date, first MTPJ interpositional arthroplasty with a PVA hydrogel implant for HR resulted in significantly improved pain and hallux dorsiflexion at an average of 14.5 months postoperatively. There was a two-year survivorship of 86.3% until the required revision to first MTPJ arthrodesis. While we report the largest single-surgeon sample to date, our results are confined to early-term outcomes after SCI. Future research should attempt to refine the indications for SCI, provide large samples of mid- to long-term outcomes, and compare SCI to other forms of interpositional arthroplasty for HR.
